# Journal of Automatic Chemistry: Editorial Board

**DOI:** 10.1155/S1463924690000104

**Published:** 1990


Journal of Automatic Chemistry, Vol. 12, No. 3 (May–June 1990), pp. 93–94

### Journal of Automatic Chemistry: Editorial Board

The following continues the *Journal's* introduction, to readers and authors, of new editorial board members.

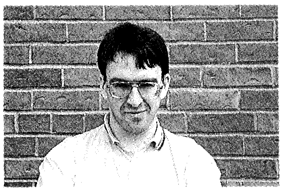

STEPHEN ROBERT BYSOUTH: After leaving Heathfield School, East Sussex in 1979, Dr Bysouth attended the University of Wales Institute of Science and Technology (UWIST), which has now become the University of Wales College, Cardiff, where he gained a B.Sc. in Environmental Science with Pure and Applied Chemistry in 1982.

During the next year and a half, he worked at Eyam Youth Hostel in Derbyshire before continuing his academic career in 1984 at the University of Technology, Loughborough, where studies of calibration techniques for atomic absorption spectroscopy and the use of flow injection methods were undertaken under the direction of Dr J. F. Tyson. During 1987, he spent three months at Fisons Pharmaceuticals whilst writing his thesis. He gained his Ph.D. in 1988 after returning to Loughborough University as research assistant to Dr Tyson studying methods of automated dilution and preconcentration for atomic spectrometry.

He has now moved to the University of Massachusetts, Amherst, Massachusetts, USA, to continue the work with Professor Tyson. Current research interests include automation of chemical analysis for improved detection limits and total automation of analysis.

He has been an associate member of the Royal Society of Chemistry, since becoming a student member in 1981, and became a member of the American Chemical Society in 1989.

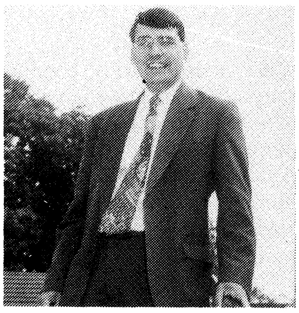

PROFESSOR LES EBDON is Deputy Director (Academic) and Professor of Analytical Chemistry at the Polytechnic South West. He is an analytical chemist of international repute, particularly in the field of analytical atomic absorption and environmental monitoring. He is the author of over 150 scientific papers and three books and has supervised more than 25 Ph.D. students. He was awarded the Royal Society of Chemistry's Silver Medal in 1986. Curently he is Chairman of the Editorial Board of the *Journal of Analytical Atomic Spectroscopy* and a member of several other editorial boards.

His research work has made a valuable contribution in relation to sample introduction into plasma systems, especially direct current plasmas. The Ebdon Nebuliser enables slurry samples to be analysed minimizing valuable operator time on sample preparation. Automated sample preparation systems, for example vapour generation and preconcentration, have also figured in his research. He has made a valuable addition in the technology transfer from academic use to industrial applications.

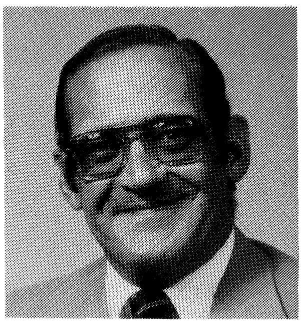

GERST ALAN GIBBON was born in Pittsburgh, Pennsylvania in 1939. He holds a B.A. degree from Albion College and the M.S. and Ph.D. from Carnegie Mellon University. From 1967 until 1974 he was Assistant Professor of Chemistry at Chatham College, Pittsburgh, Pennsylvania. Since 1974 he has been on the staff of the Pittsburgh Energy Technology Center of the US Department of Energy. His current assignment is Group Leader, Process Chemistry, Direct Coal Conversion Division.

Dr Gibbon has been active in laboratory automation and computerization for over 15 years. In particular, he has been a leading innovator in the design and implementation of Laboratory Information Management Systems (LIMS). He has given numerous presentations on such systems and has organized several symposia on the topic. He is currently President of the LIMS Institute, Inc., which is the umbrella organization for the annual International LIMS Conferences. He has also published several articles on the automation of laboratory gas chromatographs for on-line analysis.

Gerst Gibbon is a member of the American Chemical Society, Sigma Xi, The Society for Analytical Chemists of Pittsburgh, and the organizing committee of The Pittsburgh Conference on Analytical Chemistry and Applied Spectroscopy.

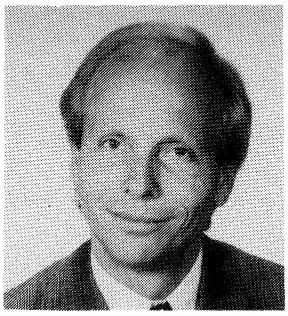

Günter Knapp was awarded his B.S. in Chemistry in 1967 from Graz University of Technology, Austria. He received his Dr techn. in Analytical Chemistry in May 1969, also from Graz University. His experience is as follows:

1971—Research fellow, AERE, Harwell, Berkshire, UK.
1974—Guest researcher, SANDOZ Research and Development Department, Basel, Switzerland.
1975—Guest researcher, Max-Planck-Institut für Metallforschung, Schwäbisch Gmünd, FR Germany.
1969–1971, 1972–1974, 1976–1979—Assistant Professor at the Department for General Chemistry, Micro- and Radiochemistry, Graz University of Technology, Austria.
1979—Associate Professor at the Department for General Chemistry, Micro- and Radiochemistry, Graz, University of Technology, Austria.
1984—Full Professor at the Department for Analytical Chemistry, Micro- and Radiochemistry, Graz University of Technology, Austria.

He has 64 publications and his research and development interests have included (1) an automatic system for the catalytic determination of trace elements; (2) mechanization in sample decomposition; (3) methods and apparatus for automatic element preconcentration; and (4) new techniques in plasma emission-spectroscopy. He is a member of the following societies: the Austrian Chemical Society, the German Chemical Society, the International Society for Trace Research in Humans, and the Austrian Society for Artificial Organs.

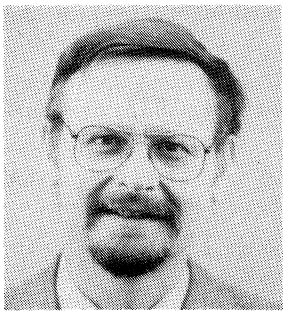

Gero Michel originally studied Physics in Basel. He then spent four years in the United States at the University of Notre Dame, South Bend, Indiana, and the University of Washington, Seattle, Washington.

Since 1970 he has worked in the Department of Laboratory Automation (recently renamed Measurement Techniques and Automation) at Ciba-Geigy in Basel, Switzerland. He is currently working in a broad field from laboratory automation to process measurement and process control.

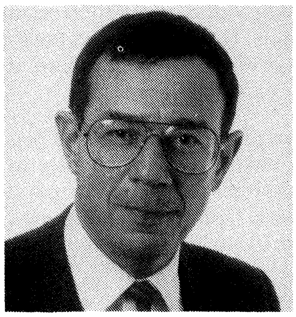

Bo Karlberg gained his B.Sc. from the University of Lund in 1967 (mathematics, chemistry, statistics) and his Ph.D. in Analytical Chemistry from the University of Umeå, Sweden in 1973 (Ion-exchange properties of the gel layer of glass electrodes). From 1973–1979 he was Manager of the 'Analytical Control Group': 18 people responsible for automation of quality control procedures at Astra Pharmaceuticals, Södertälje, Sweden (introduction of AutoAnalysers, HPLC, FIA). Then from 1979–1987 he was the Manager for Application Development, Bifok, Sweden (FIA Applications). From 1987 he has been R & D Manager at Tecator (development of analysers for water, beer, food and feed etc.). Dr Karlberg is the author of more than 40 scientific papers and one book (*Flow Injection Analysis, A practical guide*, Elsevier, 1979).

